# Proposal for a UN Peace and Development Fund: A Possible Pathway for Political and Ethical Renewal in the Modern World

**DOI:** 10.1007/s11673-024-10367-7

**Published:** 2025-01-06

**Authors:** Jeffrey D. Sachs, Paul Komesaroff

**Affiliations:** 1 Columbia University, New York, USA; 2 Monash University, Mebourne, Australia

**Keywords:** Ethics, Peace, War, Conflict, United Nations, Development, Reconciliation, Gaza, Ukraine

Testimony of Professor Jeffrey D. Sachs, University Professor at Columbia University, U.N. Security Council Session on Sustaining Peace through Common Development, November 20, 2023

*Editorial note by Paul Komesaroff: The JBI is publishing the following testimony by Professor Jeffery Sachs to the United Nations Security Council as part of our symposium on ethics in geopolitical conflicts. We are doing so because this speech eloquently articulates several critical issues relating both to the ethical basis underlying world conflicts and to the limitations of existing frameworks for responding to them.*

*Professor Sachs is the Director of the Center for Sustainable Development at Columbia University in the United States and has, over a number of decades, provided advice on economic policy and development strategies to various governments and the United Nations. In his analysis of apparently intractable conflicts in Ukraine, Gaza, Syria, and the Sahel he draws attention to the complex array of historically-conditioned factors—political, economic and cultural—from which they have arisen. He argues that if solutions are to be found they must take into account the specific circumstances of each individual conflict and the precise interplay of variables within them. There is no single principle that can be applied abstractly in every case: rather, the ethical discourses directed at finding resolutions must engage with all relevant participants and take into account the multiple values and perspectives in contention.*

*The United Nations is the pre-eminent body in the contemporary world with the authority and capacity to influence the development of conflicts and to take action to resolve them. The Security Council, as the most powerful entity within the United Nations, has extensive powers to intervene economically or even militarily to enforce conditions on nations in dispute. However, these powers are by no means unlimited, and it is well recognized that their deployment is invariably subject to the perceived self-interest of member states, especially the five permanent members of the United Nations Security Council which hold vetoes, which may limit or distort values-related objectives.*

*Using ethical arguments, Professor Sachs makes an impassioned appeal for U.N. action to establish effective peace processes in each of the four conflict areas. Without saying so explicitly, he implies that, despite the complexity of the conflicts, if ethical discourse were allowed to prevail solutions might quickly become available. Specifically, he proposes the establishment of a Peace and Development Fund that, by promoting sustainable development, would allow at least some of the factors underlying these and other conflicts to be addressed directly. He proposes that the programme be funded by wealthy states through small reductions in military outlays. The implicit vision, implicit in the entire argument, is of a reconstructed ethical dialogue that opens the way for cooperative action involving many parties in order to build peace and prosperity in place of division, enmity, and violence.*

*Readers are invited to respond to Professor Sachs’ bold proposals, in both their theoretical and their practical aspects.*

Mr. President, Ambassadors, Secretary-General Guterres, NDB President Rousseff, distinguished diplomats, ladies, and gentlemen,

My name is Jeffrey D. Sachs. I am University Professor at Columbia University. I am a specialist in the global economy and sustainable development. I appear before the U.N. Security Council on my own behalf. I represent no government or organization in the testimony I will deliver.

Today’s meeting takes place at a time of several major wars. In my testimony I will refer to four: the Ukraine War, which started in 2014 with the violent overthrow of Ukraine’s president Viktor Yanukovich; the Israel–Palestine War, which has flared repeatedly since 1967; the Syrian War, which began in 2011; and the Sahel Wars, which began in 2012 in Mali and have now spread throughout the Sahel.

These and other recent wars have claimed millions of lives, squandered trillions of dollars in military outlays, and destroyed cultural, natural, and economic wealth built over generations and indeed millennia. Wars are the worst enemy of sustainable development.

These wars may seem intractable, but they are not. Indeed, I would suggest that all four wars could be ended quickly by agreement within the U.N. Security Council. One reason is that major wars must be fed from the outside, both with external finances and armaments. The U.N. Security Council could agree to choke off these awful wars by withholding external financing and armaments. This would require an agreement among the major powers.

The other reason why these wars can end quickly is that they result from economic and political factors that can be addressed through diplomacy rather than through war. By addressing the underlying political and economic factors, the Security Council can establish conditions for peace and sustainable development. Let us consider each of the four wars in turn.

The War in Ukraine has two main political causes. The first is the attempt by NATO to expand to Ukraine despite the timely, repeated, and increasingly urgent objections by Russia. Russia considers NATO presence in Ukraine as a significant threat to Russia’s security.^1^ The second political cause is the east-west ethnic divide in Ukraine, partly along linguistic and partly along religious lines. Following the overthrow of President Yanukovych in 2014, ethnic Russian regions broke away from the post-coup government and appealed for protection and autonomy. The Minsk II agreement, endorsed unanimously by this Council in Resolution 2202, called for regional autonomy to be incorporated in Ukraine’s constitution, but the agreement was never implemented by the Government of Ukraine despite the U.N. Security Council backing.

The economic cause of the war results from the fact that Ukraine’s economy faces both west to the European Union and east to Russia, Central Asia, and East Asia. When the EU tried to negotiate a free trade agreement with Ukraine, Russia expressed alarm that its own trade and investments in Ukraine would be undermined unless a three-way agreement was reached among the E.U., Russia, and Ukraine to ensure that Ukrainian–Russian trade and investment would be sustained alongside European Union–Ukraine trade. Unfortunately, the European Union was apparently not prepared to negotiate with Russia over such a three-way arrangement, and the competing east-west orientation of Ukraine’s economy was never resolved.

This Council could end the Ukraine War quickly by addressing its underlying political and economic causes. On the political front, the P5 countries should agree to extend a security guarantee to Ukraine while also agreeing that NATO will not expand into Ukraine, thereby addressing Russia’s deep opposition to NATO enlargement. The Council should also work to achieve a lasting governance solution regarding Ukraine’s ethnic divisions. The failure of Ukraine to implement the Minsk II agreement, and of the Council to enforce the agreement, means that the solution of regional autonomy is no longer sufficient. After nearly ten years of harsh fighting, it is realistic that some of the ethnically Russian regions will remain as part of Russia, while the vast majority of Ukrainian territory will of course remain with a sovereign and secure Ukraine.

On the economics side, there are two considerations, one regarding policy and one regarding financing. On policy, Ukraine’s strong economic interest is to join the European Union while also to maintain open trade and financial relations with Russia and the rest of Eurasia. Ukraine’s trade policy should be inclusive rather than diversionary, allowing Ukraine to serve as a vibrant economic bridge between the east and west of Eurasia. On the financing side, Ukraine will need funding for reconstruction and for new physical infrastructure—such as fast rail, renewable energy, 5G, and port modernization. As I describe below, I recommend that the Security Council establish a new Peace and Development Fund, to help mobilize the financing to help Ukraine and other war zones to turn away from war towards recovery and long-term sustainable development.

Consider in a similar way the war in Israel and Palestine. Here too the war could be ended quickly by the Council enforcing the many U.N. Security Council resolutions made over several decades calling for a return to the 1967 borders, an end of Israel’s settlement activities in occupied territories, and the two-state solution, including UNSC resolutions 242, 338, 1397, 1515, and 2334. It is clear that Israel and Palestine are unable to reach bilateral agreements in line with these U.N. Security Council resolutions. On both sides, hardliners repeatedly frustrate moderates who seek peace based on the two-state solution.

It is high time, therefore, for the U.N. Security Council to enforce its decisions, by implementing a just and lasting solution that is in the interests of both Israel and Palestine, rather than allowing hardliners on both sides to ignore the mandate of this Council and thereby to threaten the global peace. My recommendation to this Council is that it immediately recognize the State of Palestine, in a matter of days or weeks, and welcome Palestine as a full member of the United Nations, with capital in East Jerusalem and with sovereign control over the Islamic Holy Sites. The Council should also establish a peacekeeping force, drawn heavily from the neighbouring Arab countries, to help provide security in Palestine. Such an outcome is the overwhelming will of the international community, and in the manifest interest of both Israel and Palestine, despite the vociferous objections by hardline rejectionists on both sides of the divide.

As with the case of Ukraine, the failure of this Council to enforce its earlier resolutions regarding Israel and Palestine have made the current situation far more difficult to resolve. Israel’s illegal settlements have by now expanded to more than 600,000 settlers. Yet Israel’s brazen and long-standing violation of the U.N. Security Council in this regard is no reason for the Council to shrink from decisive action now, especially as Gaza is in flames, and the broader region is a tinderbox that could explode at any moment.

An economic strategy should accompany the political strategy. Most importantly, the new sovereign State of Palestine must be economically viable. This will require several economic measures. First, Palestine should benefit from offshore oil and gas deposits in Palestine’s territorial waters. Second, the new Peace and Development Fund should help Palestine to finance a modern port in Gaza and a secure road and rail link that connects Gaza and the West Bank. Third, the vital water resources of the Jordan Valley must be equitably shared between Israel and Palestine, and both nations together should be supported to secure a substantial increase in desalinization capacity to meet the urgent and growing water needs of both countries. Fourth, and most importantly, both Israel and Palestine should become part of an integrated sustainable development plan for the Eastern Mediterranean and Middle East that supports climate resilience and the region’s transition to green energy.

The Council can similarly end the war in Syria. The Syrian War broke out in 2011 when several regional powers and the United States joined forces to topple the government of Syrian President Bashar al-Assad. This deeply misguided regime-change operation failed but triggered a prolonged war with enormous bloodshed and destruction, including of ancient cultural heritage sites. The Council should make clear that all P5 countries and the countries in Syria’s neighbourhood are in full agreement that all regime-change attempts are now permanently ended and that the U.N. Security Council intends to work closely with the Syrian Government on reconstruction and development.

On the economic side, Syria’s best hope is to become closely integrated in the Eastern Mediterranean—Middle East region, especially through the construction of physical infrastructure (roads, rail, fibre, power, water) connecting Syria with Turkey, the Middle East, and the Mediterranean nations. As with Israel and Palestine, this investment programme should be partly funded by a new Peace and Sustainable Development Fund created by this Council.

The war in the Sahel has similar roots to the war in Syria. Just as regional powers and the U.S. aimed to overthrow the regime of Bashar al-Assad in 2011, the major NATO powers similarly aimed to overthrow the regime of Moammar Qaddafi in Libya in 2011. In pursuing this aim, they grossly exceeded the mandate of U.N. Security Council Resolution 1973, which had authorized the protection of Libya’s civilian population but certainly not a NATO-led regime change operation. The violent overthrow of the Libyan government quickly spilled over to the impoverished countries of the Sahel. Poverty alone made these Sahelian countries highly vulnerable to the influx of armaments and militias. The result has been ongoing violence and multiple coups, gravely undermining the possibility of economic improvement.

The Sahel crisis today is first and foremost a crisis of insecurity and poverty. The Sahel is a region that is semi-arid to hyper-arid, with chronic food insecurity, hunger, and extreme poverty. Most of the countries of the region are landlocked, causing massive difficulties for transport and international trade. Yet at the same time, the region has massive deposits of highly valuable minerals, great biodiversity and agronomic potential, huge solar energy potential, and of course an enormous human potential that is not yet realized because of a chronic shortfall of schooling and training.

The countries of the Sahel form a natural aggregation for regional economic investment in infrastructure. The entire region urgently needs investments in electrification, digital access, water and sanitation, and road and rail transport, as well as in social services, notably education and healthcare. As the Sahel is among the poorest regions of the world, the governments are utterly unable to finance the needed investments. Here too, and perhaps more than in any other region, the Sahel needs external funding to make the transition from war to peace, and from extreme poverty to sustainable development.

All P5 members, and indeed the whole world, suffer adverse consequences from the continuation of these wars. All are paying a price in terms of financial burdens, economic instability, risks of terrorism, and risks of a wider war. The Security Council is in a position to take decisive actions to end the war precisely because it is clear that the interest of all U.N. Security Council members, and notably all of the P5 countries, is to bring these long-standing wars to an end before they escalate into even more dangerous conflicts.

The Security Council is vested with considerable powers by the U.N. Charter when it has the resolve of its members. It can introduce peacekeepers, and even armies if necessary. It can impose economic sanctions on countries that do not comply with UNSC Resolutions. It can provide security guarantees to nations. It can make referrals to the International Criminal Court to stop war crimes. In short, the Council is certainly able to enforce its resolutions if it chooses to do so. For the sake of global peace, let the Council now choose to end these wars.

The U.N. Security Council should also bolster its toolkit by engaging in economic peacebuilding alongside the more usual decisions on borders, peacekeepers, sanctions, and the like. I have mentioned several times the idea of creating a new Peace and Development Fund that the U.N. Security Council could deploy to create positive dynamics for sustainable development, and to encourage other investors—such as the World Bank, the IMF, and the regional Multilateral Development Banks—to co-invest in peace-making.

I would recommend three guideposts for such a new fund.

First, it would be funded by the major powers by transferring a part of their military outlays to global peace-making. The U.S., for example, now spends roughly $1 trillion per year on the military, while China, Russia, India, and Saudi Arabia are the next biggest spenders, with combined military outlays that are a bit more than half of the U.S., perhaps around $600 billion. Suppose that these countries reduced military outlays by just 10 per cent and redirected the savings to the Peace and Development Fund. That alone would free up around $160 billion per year. Even that sum could be leveraged with some financial engineering to enable annual loans of say $320 billion per year, that is, enough to help today’s war zones to begin a vigorous turn to recovery and development.

Second, the fund would emphasize regional integration. This is paramount for peace-making as well as for successful development. Ukraine would be helped to integrate both west (to the E.U.) and east (toward Russia, Central Asia, and East Asia). Israel, Palestine, and Syria would all be helped to integrate in an infrastructure network for the EMME region, deepening peace as well as economic development. The Sahel countries would be helped to break their isolation and lack of basic services through a network of infrastructure for roads, rail, ports, fibre, and power.

Third, the Peace and Development Fund would partner with other funding streams, such as China’s Belt and Road Initiative, the E.U.’s Global Gateway, the G7’s Global Partnership for Infrastructure and Investment, and increased lending by the Bretton Woods institutions and the regional development banks. Interestingly, the Fund for Peace and Development could be a vehicle for greater investment partnerships linking China, the E.U., the United States, and the G7. This too would be a contribution towards peace, not only into today’s war zones but also among the world’s major powers.

Directly across the street from us is Isaiah’s wall, with the visionary words of the great Jewish prophet of the 8th century BCE: “They shall beat their swords into ploughshares, and their spears into pruning hooks; nation shall not lift up sword against nation, neither shall they learn war anymore.” It is time to honour Isaiah’s words by ending these useless wars, slashing military outlays and turning the savings into new investments in education, healthcare, renewable energy, and social protection.

The proposal to redirect today’s military outlays into tomorrow’s sustainable development finance builds not only upon Isaiah’s enduring wisdom but on the proposals of religious leaders and the world’s nations in the U.N. General Assembly. Pope Paul VI in his brilliant encyclical *Populorum Progresio* (1967) called on world leaders “to set aside part of their military expenditures for a world fund to relieve the needs of impoverished peoples.” The U.N. General Assembly took up this cause in UNGA Resolution 75/43, calling on “the international community to devote part of the resources made available by the implementation of disarmament and arms limitation agreements to economic and social development, with a view to reducing the ever-widening gap between developed and developing countries.”

As an American, I am proud that our greatest President, Franklin Delano Roosevelt, was the visionary who oversaw the establishment of this great institution. I believe firmly in the capacity of the United Nations, and of this Security Council, to keep the peace and to promote sustainable development. When all 193 U.N. member states, or 194 with the membership of Palestine, live up to the U.N. Charter, we will have a new Global Age of Peace and Sustainable Development.

## Footnotes

1 We may recall that the U.N. Charter article 2(4) prohibits not only the use of force but also the threat thereof.

